# Genetic and Functional Diversity of Bacterial Microbiome in Soils With Long Term Impacts of Petroleum Hydrocarbons

**DOI:** 10.3389/fmicb.2018.01923

**Published:** 2018-08-22

**Authors:** Anna Gałązka, Jarosław Grządziel, Rafał Gałązka, Aleksandra Ukalska-Jaruga, Joanna Strzelecka, Bożena Smreczak

**Affiliations:** ^1^Department of Agriculture Microbiology, Institute of Soil Science and Plant Cultivation, State Research Institute, Puławy, Poland; ^2^Department of Soil Science Erosion and Land Protection, Institute of Soil Science and Plant Cultivation, State Research Institute, Puławy, Poland

**Keywords:** bacterial community, genetic diversity, metabolic profiles, Biolog EcoPlates, V3–V4 16S rRNA gene region, NGS

## Abstract

Soil contamination with petroleum, especially in the area of oil wells, is a serious environmental problem. Restoring soil subjected to long-term pollution to its original state is very difficult. Under such conditions, unique bacterial communities develop in the soil that are adapted to the contaminated conditions. Analysis of the structure and function of these microorganisms can be a source of valuable information with regard to bioremediation. The aim of this study was to evaluate structural and functional diversity of the bacterial communities in soils with long-term impacts from petroleum. Samples were taken from the three oldest oil wells at the Crude Oil Mine site in Węglówka, Poland; the oldest was established in 1888. They were collected at 2 distances: (1) within a radius of 0.5 m from the oil wells, representing soil strongly contaminated with petroleum; and (2) 3 m from the oil wells as the controls. The samples were analyzed by 16S rRNA sequencing and the community level physiological profiling (CLPP) method in order to better understand both the genetic and functional structure of soil collected from under oil wells. Significant differences were found in the soil samples with regard to bacterial communities. The soils taken within 0.5 m of the oil wells were characterized by the highest biodiversity indexes. *Alphaproteobacteria*, *Betaproteobacteria*, *Gammaproteobacteria* were strongly correlated with biological activity in these soils. Families of *Alphaproteobacteria* were also dominant, including: *Bradyrhizobiaceae, Rhizobiaceae, Rhodobacteraceae, Acetobacteraceae, Hyphomicrobiaceae*, and *Sphingomonadaceae.* The study showed that the long term contamination of soil changes bacterial communities and their metabolic activity. Even so, natural bioremediation leads to the formation of specific groups of bacteria that actively grow at the site of contamination in the soil.

## Introduction

A constantly increasing pollution of soil, air and water, by processes such as industrial activity, low efficiency of metal recovery methods, and agricultural chemicalization, poses a major impact to the health of humans and to nature, in general (Semple et al., 2006; Child et al., 2007; Zhong et al., 2011). With regard to soil pollution, it is not only necessary to create an efficient monitoring system but also to develop economical and efficient techniques for treatment and immobilization of toxic compounds at the place of their deposition, thus preventing more widespread environmental pollution (Gałązka et al., 2012; Nwaichi et al., 2015; Silva et al., 2015). There are a number of technologies that enable the deactivation or removal of toxic substances from a substrate, in most cases based on physicochemical extraction methods (Sutton et al., 2013; Gałązka and Grządziel, 2016). Unfortunately, their application is associated with extremely high costs and a complete elimination of soil microorganisms. The restoration of biological activity in these areas is very difficult and almost always requires human intervention (Doong and Lei, 2003; Khan, 2005; Vazquez et al., 2013). Therefore, rebuilding near-natural ecosystems in such cases is an extremely long and expensive process.

Long-term soil pollution in oil fields is a serious environmental problem and restoring the soil to its original state is very difficult. Under such conditions, unique bacterial communities adapted to the contamination conditions develop in the soil. Long-term contamination causes an accumulation of a variety of petroleum products in the soil, including both polycyclic aromatic hydrocarbons (PAHs) and aliphatic hydrocarbons. It negatively influences both biodiversity of microorganisms and soil function. It is especially visible in the areas of oil wells, where the accumulation has been going on for many years. Such constantly contaminated soil has no chance for effective remediation; however, even in these unfavorable conditions there are groups of active microorganisms that are able to dwell in the soil. Decomposition of complex mixtures, such as petroleum can be performed by mixed microorganism cultures (microbial complexes) with diverse activities and the ability to use hydrocarbons as a source of carbon and energy (Hartmann and Widmer, 2006; Meng et al., 2011; Gałązka and Gałązka, 2015). Typically, biodegradation of crude oil derivatives occurs via cometabolism, which consequently plays a very important role in the bioremediation process. The hydrocarbons are not a source of carbon and energy in this case, but they are co-substrates, with their degradation occurring sequentially by the participation of different groups of microorganisms. At present, cometabolism is considered to be one of the most important mechanisms in the transformation of PAHs in soil (Doong and Lei, 2003). For example, parathion is cometabolized by *Pseudomonas stutzeri* to 4-nitrophenol and diethylphosphate, and phenol is then used as a source of carbon and energy by *P. aeruginosa*. Often, phenol or toluene is used as a co-substrate for compounds resistant to biodegradation. However, this does not mean, that in order to achieve an effective biodegradation process, one only has to use a co-substrate and the most active strains. The situation is more complex, as it is often necessary to introduce bacteria which are almost inactive during decomposition, but may facilitate the enzymatic activity of other species (Dellagnezze et al., 2016). Enzymes synthesized by individual strains of bacteria can provide for hydroxylation, oxidation, denitrification, deamination, hydrolysis or acylation reactions that complement each other in the formation of complete pathways of contamination mineralization (Child et al., 2007; Naether et al., 2012; Chen et al., 2017).

Therefore, it is very important to know precisely the genetic structure and function of microorganisms at the site of contaminations, both for a greater understanding of the processes of bioremediation and as a source of inoculum in managing such areas (Gomez et al., 2004; Mishra and Nautiyal, 2009; Peng et al., 2015). Re-establishing soil microbial communities is essential as they are responsible for physiological and metabolic processes of great importance for soil quality (Ranjard et al., 2003; Bundy et al., 2009; Lauber et al., 2009; Rutgers et al., 2016). Studies of bacterial communities in contaminated soil are enhanced by recent advances in genomics, transcriptomics and proteomics. Included in genomic methods are functional bacterial fingerprinting (Biolog EcoPlates System) and Next Generation Sequencing (NGS). NSG of hypervariable regions, such as in 16S rRNA genes from bacteria, allows one to determine the genetic diversity of microorganisms within a population without the need for cell culture (Xu, 2010; Malla et al., 2018; Pichler et al., 2018). The PCR-based method has been successfully applied to identify microorganisms acting as pollutant degraders in soil, such as naphthalene, salicylate or benzoate degraders from the class β-*proteobacteria* (Friedrich, 2006; Kozich et al., 2013; Sarkar et al., 2016). In addition to determining the diversity of microorganisms, metagenomics analysis can also be used to search for functional genes with regard to pollutant degradation, and so proof that a specific metabolic activity is occurring among the members of the microbiome (Bartram et al., 2011; Rosselli et al., 2016). In order to obtain as much information as possible about functional and genetic structure of soils in this study, it was decided to use 2 methods: the NGS technique (V3–V4 16S rRNA gene region), and the community level physiological profiling (CLPP) method.

The importance of evaluating bacterial functional and structural diversity in soil directly from the contaminated site is that you are defining a natural bioremediation process (Hartmann and Widmer, 2006). Unfortunately, there is still a lack of relevant indexes to assess soil quality. So far, although a number of indicators have been used to evaluate non-agricultural soil quality, no universal formula has been developed (Wyszkowska and Kucharski, 2005; Bastida et al., 2006, 2008; Dawson et al., 2007; Wang et al., 2010; Wyszkowska et al., 2015). Included among available indicators is the soil quality index, defined as the smallest set of soil parameters that can provide information about soil quality and its ability to perform certain functions (Nannipieri et al., 2003). The parameters of this index are: pH, organic matter content, microbial biomass, enzymatic activity, and respiratory performance. One of the more well-known quality indexes is the microbiological degradation (MD) Index, which takes into account the following parameters: semiarid degraded soils, dehydrogenase, urease activities, respiration, water-soluble carbon, and water-soluble carbohydrates (Bastida et al., 2006). Another index is the Soil Quality Index (Dawson et al., 2007). Microbial biomass carbon, dehydrogenase activity, seed germination, respiration, and earthworm toxicity were the parameters used in this case. Chen et al. (2017) presented the Integrated Pollution (IP) Index, which included measures of several heavy metals. So far, none of these sets of parameters have been identified as a universal molecular indicator of soil quality. Such indicators could be extremely useful in determining the role of specific microorganisms in the soil and the selection of key enzymes related to improving the functioning of the soil (Ros et al., 2008).

The aim of this study was to evaluate the functional and structural diversity of bacterial communities in soils with long-term impacts from contamination with petroleum. The assessment was conducted on the basis of distance from direct contamination (soil taken directly from oil wells and from 3 m distance). The research hypothesis assumes that significant changes will be observed in the functional and structural diversity of microorganisms between the soils. Determining the function and quality of bacteria in contaminated soil is the basis for further research to select the strains active in bioremediation. Such bacteria could then be used in the industry in future bioremediation processes.

## Materials and Methods

### Soil Samples

**FIGURE 1:**
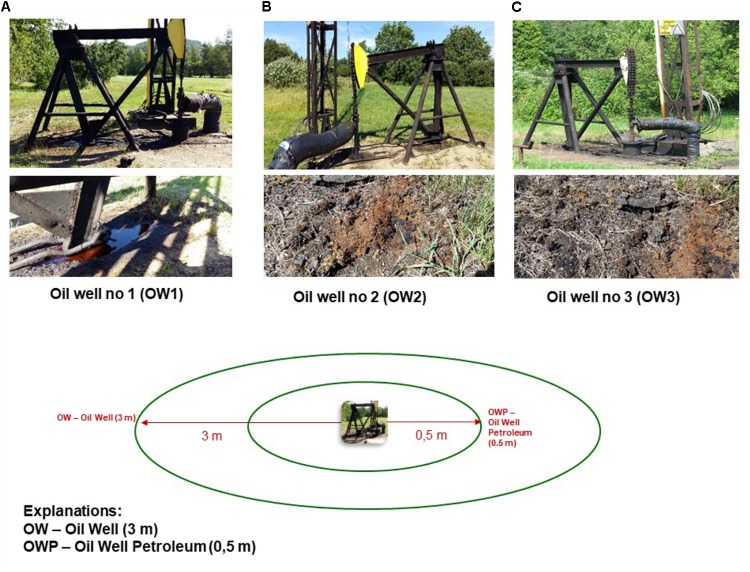
Oil wells at the Crude Oil Mine in Węglówka, Poland. **(A)** Oil well no. 1; **(B)** Oil well no. 3; **(C)** Oil well no. 3.

Soil samples were collected in July 2017 according to the methods of Polish Standard (1998). The soils (light loamy sand – soil texture Casagrande’a method) were taken from the area of oil wells in Węglówka near Krosno (Podkarpackie Voivodeship, Poland). The soils had been contaminated with petroleum over the long-term. Samples were taken from the 3 oldest oil wells at the Crude Oil Mine site in Węglówka, Poland, the oldest of which dated from 1888 (**Figure 1**). The Global Positioning System (GPS) locations of the oil wells are given in **Table 1**. Soil samples were collected at two distances: within a radius of 0.5 m of the oil wells (OWP – Oil Well Petroleum; OWP1, OWP2, and OWP3) and at a distance of 3 m from the oil wells (OW – Oil Well; OW1, OW2, and OW3). The soil samples were taken from the 0–20 cm layer in three replicates and passed through a 2 mm sieve. The samples were then stored in a refrigerator (4°C) until they were analyzed. The basic chemical, biochemical and microbiological properties in contaminated soil were determined. The basic chemical properties of the soils were marked: pH (PN-ISO 10390:1997), total organic carbon (C_org_-using the Tiurin’s method) and total Kjeldahl nitrogen content (*N*_total_-using flow spectrometry, wet sample mineralization). In addition, the total content of petroleum hydrocarbons, and the PAH levels were determined by gas chromatography, while the content of trace elements after microwave treatment with aqua regia were assayed using inductively coupled plasma mass spectrometry (ICP-MS). Moreover, the functional and genetic bacterial diversities were analyzed.

**Table 1:** The location of soil samples and archive designations for the deposited sequencing data.

Sample ID	GPS location	Location	Environment	Sequence Read Archive (SRA) NCBI ID
OW1	49°76′60′′N, 21°79′69′′E	Oil well no. 1, Węglówka, Poland	Grassland soil	SRX4084526
OW2	49°76′90′′N, 21°77′60′′E	Oil well no. 2, Węglówka, Poland	Grassland soil	SRX4084524
OW3	49°76′80′′N, 21°77′45′′E	Oil well no. 3, Węglówka, Poland	Grassland soil	SRX4084528
OWP1	49°76′60′′N, 21°79′69′′E	Oil well no. 1, Węglówka, Poland	Soil under oil well	SRX4084525
OWP2	49°76′90′′N, 21°77′60′′E	Oil well no. 2, Węglówka, Poland	Soil under oil well	SRX4084523
OWP3	49°76′80′′N, 21°77′45′′E	Oil well no. 3, Węglówka, Poland	Soil under oil well	SRX4084527

*Public data was deposited in the Sequence Read Archive (SRA) NCBI: https://www.ncbi.nlm.nih.gov/sra/SRP146074. OWP, soil sample taken within a radius of 0.5 m; OW, soil sample taken at a distance of 3 m from the oil well*.

### Determination of PAH Levels

The analysis of PAHs comprised 16 individual compounds from the Ual compounds from the US EPA list. Soil samples were sieved to obtain a grain size ≤ 0.10 mm and spiked with 10 μl of internal standard solution containing 5 deuterated PAHs: d8-naphtalene, d10-acenaphtene, d10-phenanthrene, d12-chrysene and d12-perylene, each at a concentration of 100 μg cm^3^. Samples were extracted with dichloromethane in an Accelerated Solvent Extractor (ASE200, Dionex Co., Sunnyvale, CA, United States); extraction temperature 100°C, static time 5 min, and pressure 1200 psi. The extracts were concentrated in 1 ml hexane, cleaned up on glass columns filled with 1 g activated silica gel suspended in dichloromethane, and finally eluted with 5 ml CH_2_Cl/n-hexane (2/3, v/v). PAH levels were determined by Triple Quadrupole Gas Chromatograph-Mass Spectrometer (GC-MS/MS) on an Agilent 7890B GC system (Agilent Tech., Santa Clara, CA, United States), equipped with an Agilent 7000C detector and Agilent 7693 Autosampler. PAH resolution was achieved on a HP-5 MS fused capillary column with film thickness of 0.25 μm, at a 250°C splitless injection system temperature with helium as a carrier gas. Data were collected in Multiple Reaction Monitoring (MRM) mode. The certified reference material (CRM 131), laboratory control sample and solvent blank sample procedure were used for quality assurance and quality control (OA/OC). The precision expressed as a relative standard deviation (RSD) was in the range of 5–12% and the recovery for individual compounds from CRM 131 was within 62–84%. The limit of quantification (LoQ) for individual PAH compounds ranged from 0.02–2.10 μg kg^-1^, while the limit of detection (LoD) fitted within the 0.01–0.81 μg kg^-1^ range.

### Extraction of Petroleum Hydrocarbons

Five grams of dry soil with grain size ≤ 0.10 mm were extracted with 120 cm^3^ petroleum ether in an automated Soxhlet apparatus (Buchi Universal Extraction System, Buchi 811) for 35 cycles at 40–60°C. Extracts were collected in glass vials and evaporated on a vacuum rotary evaporator at 40°C to near dryness. The glass vials were then left open under a flow hood to remove traces of ether. The concentration of petroleum hydrocarbons expressed as gkg^-1^ of soil dry mass was evaluated from the weight of the concentrated hydrocarbon extract. The extractions were carried out in triplicate. For quality control a solvent blank was included for each analytical series.

### Determination of Trace Element Content in Soil Samples – ICP-MS Technique

Soil samples were digested in aqua regia with involvement of middle pressure (32 bars) microwave digestion system - Mars Xpress from CEM Corp., Matthews, NC, United States. Quantitative analysis of metals content were conducted on ICP-MS 7500ce instrument from Agilent Technologies, Santa Clara, CA, United States. The 0.5 g of air dried soil sieved through 2 mm mesh and grinded on mortar grinder were used for the digestion. Aqua regia was prepared from Instra – Analyzed grade hydrochloric and nitric acids from J.T. Baker Chemical Co, Phillipsburg, NJ, United States. After digestion the solution was transferred to falcon vial and diluted to 50 ml with 0.05 μS/cm distilled water. Prior to ICP-MS analysis, samples were diluted 10 times. Exactly the same procedure was performed for blanks and certified reference materials. To minimize the matrix effect and ensure long term stability, analysis were conducted in presence of 45Sc, 89Y, 159Tb as internal standards. Accuracy of the method was 10% and quantification limits were 0.01 mg kg^-1^. Detailed information about methods was described in paper Gałązka and Gałązka (2015).

### CLPP Analysis Using Biolog EcoPlates

The CLPP was evaluated using Biolog EcoPlates (Biolog Inc., Hayward, CA, United States) with 31 different carbon sources (Pohland and Owen, 2009). Soil suspension for the inoculation of wells in microplates was prepared as follows. One gram of soil was weighed and transferred to a conical flask holding 99 cm^3^ sterile 0.9% NaCl, vortexed for 30 min at 150 rpm and at 25°C, after which the samples were cooled for 30 min to 4°C. After that, 120 mm^3^ was transferred to each well of an EcoPlate and incubated in the dark at 25°C for 216 h. The experiment included three replications. The results were read on a MicroStation ID system (Biolog Inc., Hayward, CA, United States). The extent to which carbon sources were used was determined through the reduction of colorless tetrazolium chloride (TTC) to red triphenyl-formasane (TPF) (Islam et al., 2011; Gałązka et al., 2017). Intensity of color development was recorded at = 590 nm for a period of 2016 h at 24-h intervals. The most intensive metabolism of carbon substrates was observed after 72–120 h of incubation. The activities of soil microorganisms are based on all carbon sources and on grouped sources defined as amines and amides, amino acids, carbohydrate, carboxylic acid and polymers (Rutgers et al., 2016). The results were expressed as Average Well-Color Development (AWCD) and the Shannon–Wiener (*H*′), Simpson (*D*), Richness (*R*), Evenness (*E*) indices.

### DNA Extraction, NGS and Bioinformatics

The FastDNA SPIN Kit for Soil (MP Biomedicals, Solon, OH, United States) was used to extract total DNA from 0.5 g of soil. The concentration and quality of the extracted DNA was determined using NanoDrop 2000 spectrophotometer (Thermo Fisher Scientific, Wilmington, DE, United States).

Metagenomic analysis was conducted based on the hypervariable region V3–V4 of the 16S rRNA gene. Specific primers 341F and 785R were used for amplification of this region and library preparation. PCR reaction was conducted with the Q5 Hot Start High-Fidelity DNA Polymerase kit (NEB Inc., Ipswich, MA, United States) with reaction conditions according to manufacturer’s specifications. Sequencing was conducted on a MiSeq sequencer in 2 × 250 bp paired–end (PE) technology using the v2 Illumina chemistry kit. The reactions were carried out according to the Illumina V3–V4 16S RNA amplification protocol (Illumina, San Diego, CA, United States) and sequencing was performed on an Illumina MiSeq PE300 (Genomed S.A., Warsaw, Poland). Automatic data analysis was performed on MiSeq and in Cloud environment BaseSpace by Illumina, using the 16S Metagenomics protocol (ver. 1.0.1). The libraries were prepared in an analogous way to the attached Illumina protocol.

From dereplicated fastq files to remove redundancy, amplicon sequence variants (ASVs) were extracted using the DADA2 version 1.8 package (Callahan et al., 2016) in R version 3.4.3 (R Core Team, 2016) with the following parameters: *filterAndTrim* was used, based on quality plots; the forward and reverse sequences were trimmed to 250 bp; and the first-left 20 bp were removed (containing primers and low-quality bases) from both direction reads. Filtering of sequences was set to: *maxN* = 0, *maxEE* = 5 (for both reads), *truncQ* = 2; where *maxN* was maximum number “*N*” bases, *maxEE* corresponded to maximum expected errors calculated from the quality score (*EE* = sum [10ˆ(-Q/10)] and truncQ parameter truncated reads at the first instance of a quality score ≤ 2. Other parameters were set to default. The error rates were estimated by *learnErrors*, where the *nbases* parameter was set to 10ˆ8. Sequences were dereplicated using *derepFastq* with default parameters and exact sequence variants were resolved using *dada*. Next *removeBimeraDenovo* was used to remove chimeric sequences, applying the consensus method. At this step, 0.047% sequences were identified as chimeric and removed.

Taxonomy was assigned against the latest version of RDP database. RDP taxonomic training data was formatted for DADA2 (RDP trainset 16/release 11.5), using Naïve Bayesian Classifier (Wang et al., 2007) implemented in *assignTaxonomy*, setting *minBoot* parameter to 50. Sequences classified as mitochondrial and chloroplasts were filtered out using the *subset_taxa* function in the *phyloseq* package (McMurdie and Holmes, 2013). The resulting taxa table was agglomerated accordingly to each taxa level using *tax_glom* and unclassified reads were retained for statistical purposes. Next, the taxa abundances were transformed into percentages.

The basic sequence information was deposited in the Sequence Read Archive (SRA), NCBI (**Table 1**).

The software package Statistica 10.0 (Statsoft Inc., United States) were used to statistical analyses performed. Collected data was subjected to analysis of variance (ANOVA) for the comparison of means. In addition, significant differences were calculated according to Tukey’s HSD *post hoc* test at *P* < 0.05 significant levels. The AWCD was evaluated according to Garland and Mills (1991) in accordance with formula AWCD = Σ (*C*-*R*)/95; where *C* was the absorbency in each well, and *R* was the absorbency in the control well. The Shannon–Wiener (*H*′) index was evaluated in accordance with formula *H*′ = -Σpi(lnpi), pi was the ratio of the absorbance of each well to the absorbency of all wells (Gomez et al., 2006). The Simpson (*D*) index was evaluated in accordance with the formula: *D* = 1-(Σ*n* (*n*-1)/*N*(*N*-1), where, *n* = number of individuals of each species, *N* = total number of individuals of all species. The Tukey’s range test was used to identify homogeneous groups at the significance level *P* = 0.01. The obtained results were also submitted to principal component analysis (PCA) in order to determine the common relations between the bacterial core metagenome and soils collected from different oil wells. Permutational multivariate analysis of variance (PERMANOVA) was used to compare the bacterial diversity between soils taken from different sites. This was performed with 999 permutations using the Adonis function of the PAST package (v 3.16).

## Results

Evaluation of functional and structural diversity of bacteria in soil contaminated with petroleum long-term was based on two methods, as a means for establishing parameters for determining soil quality: the NGS technique (V3–V4 16S rRNA gene region), and the CLPP method.

### Chemical Analysis of Soil Samples

Soils contaminated with petroleum were characterized by pH in the range from 4.75 (OWP3) to 5.54 (OW3; **Table 2**). The organic carbon (C_org_) content was in the range from 3.07% (OWP1) to 6.07% (OW1). The soil taken from oil well no 1 (OWP1) was characterized by the highest content of PAHs (Σ16 PAHs = 3.062 mg⋅kg^-1^; **Table 2**). The Σ16 PAH’s in other soil samples ranged from 1.885 mg⋅kg^-1^ (OW3) to 2.938 mg⋅kg^-1^ (OW1). The samples taken directly from oil wells were characterized by higher contents of PAHs compared to the samples taken from 3 m distance (except for sample OW1). Also these soils were characterized by a higher content of trace elements (**Table 3**). The content of individual metals in the soils varied and depended on the collection site and the distance from the oil well. The highest content of Mn (896.1 mg⋅kg^-1^) was observed in soil sample OW1. Similar results were observed in the case of sodium (Na) and calcium (Ca) (**Table 3**).

**Table 2:** Characteristics of soils contaminated with petroleum in terms of PAH and aliphatic hydrocarbon content, pH, *C*_org_, and *N*_tot_.

Parameters	Sample ID					
	OW1	OW2	OW3	OWP1	OWP2	OWP3
pH_H2O_	5.43^b^	5.23^b^	5.54^b^	4.75^a^	5.10^a^	4.90^a^
*C*_org_ (%)	6.07^d^	5.65^d^	5.21^c^	3.07^a^	4.11^b^	3.18^a^
*N*_tot_ (%)	0.156^b^	0.222^c^	0.162^b^	0.036^a^	0.117^b^	0.162^b^
*C*/*N*	38.91^b^	25.45^c^	32.16^b^	85.28^a^	35.13^b^	19.63^c^
**PAH’s [mg kg^-1^]**		
Naphthalene	0.090^b^	0.053^b^	0.092^b^	0.124^a^	0.189^a^	0.122^a^
Acenaphthylene	0.039^b^	0.060^b^	0.060^b^	0.166^a^	0.097^a^	0.140^a^
Acenaphthene	0.037^b^	0.051^b^	0.055^b^	0.144^a^	0.084^a^	0.121^a^
Fluorene	0.048^b^	0.059^b^	0.064^b^	0.163^a^	0.106^b^	0.138^a^
Anthracene	0.132^b^	0.067^c^	0.102^c^	0.003^b^	0.146^b^	0.143^b^
Phenanthrene	0.126^b^	0.073^c^	0.015^c^	0.007^b^	0.143^b^	0.179^b^
Fluoranthene	0.253^b^	0.100^d^	0.173^c^	0.245^b^	0.134^c^	0.183^c^
Pyrene	0.258^b^	0.107^d^	0.180^c^	0.355^b^	0.150^c^	0.191^c^
Chrysene	0.238^b^	0.080^d^	0.132^c^	0.002^b^	0.141^c^	0.147^c^
Benz[a]anthracene	0.245^a^	0.086^c^	0.137^c^	0.373^a^	0.152^c^	0.120^c^
Benzo[b]fluoranthene	0.291^d^	0.100^b^	0.145^c^	0.317^e^	0.164^c^	0.210^d^
Benzo[k]fluoranthene	0.228^d^	0.078^b^	0.139^c^	0.002^e^	0.091^c^	0.206^d^
Benzo[a]pyrene	0.093^b^	0.077^b^	0.091^b^	0.202^c^	0.116^c^	0.170^c^
Indeno[1,2,3-cd]pyrene	0.097^c^	0.109^b^	0.115^b^	0.306^d^	0.173^b^	0.253^d^
Dibenz[a,h]anthracene	0.261^b^	0.118^c^	0.154^c^	0.297^b^	0.171^c^	0.250^b^
Benzo[g,h,i]perylene	0.501^b^	0.298^c^	0.232^c^	0.355^a^	0.199^e^	0.298^c^
Σ16 PAH’s	2.938^b^	1.517^c^	1.885^c^	3.062^b^	2.254^d^	2.870^d^
**Aliphatic hydrocarbons [g kg^-1^]**		
Aliphatics	37.9^c^	35.3^c^	13.9^b^	26.4^c^	2.3^a^	10.0^a^

*The results are shown as the mean of three repetitions (n = 3). OWP, soil sample taken within a radius of 0.5 m; OW, soil sample taken at a distance of 3 m from the oil well. Treatment means separated by different letters are significantly differ (Tukey’s mean separation test, *P* < 0.05)*.

**Table 3:** Characteristics of soils contaminated with petroleum in terms of trace element content.

	OW1	OW2	OW3	OWP1	OWP2	OWP3
**Trace elements content [mg kg^-1^]**		
Li	14.23^a^	18.51^b^	24.34^b^	4.88^c^	13.93^a^	5.95^c^
Cr	22.26^c^	36.79^b^	44.28^b^	10.49^a^	34.45^b^	16.76^a^
Mn	896.10^a^	743.66^a^	615.33^a^	221.25^c^	593.22^b^	214.24^c^
Co	5.28^b^	8.10^a^	8.33^a^	2.28^c^	6.52^b^	2.79^c^
Ni	17.67^b^	24.79^b^	29.81^a^	9.65^c^	27.82^a^	10.57^c^
Cu	28.23^c^	24.56^c^	38.13^b^	7.86^d^	45.56^b^	16.60^c^
Zn	66.30^a^	107.62^a^	79.14^a^	26.27^c^	493.54^b^	502.59^b^
Mo	0.74^b^	0.73^b^	0.94^b^	0.35^b^	2.65^a^	0.62^b^
Cd	0.25^b^	0.39^b^	0.22^b^	0.08^a^	0.20^b^	0.37^b^
Sn	4.80^b^	4.52^b^	2.65^b^	0.51^c^	2.28^b^	1.32^c^
Pb	36.91^b^	28.87^b^	22.92^b^	24.04	31.50^b^	28.60^b^
Na	791.30^b^	241.10^c^	584.44^b^	155.80^c^	1001.78^a^	132.15^c^
**[g kg^-^**^1^]		
Mg	4.35^a^	3.73^a^	4.31^a^	1.54^b^	3.15^a^	1.76^b^
Al	16.79^b^	23.84^a^	27.35^a^	5.95^c^	12.18^b^	7.39^c^
K	3.17^b^	6.12^a^	7.30^a^	1.95^c^	2.75^b^	1.97^c^
Ca	29.46^a^	4.71^b^	19.34^a^	7.83^b^	17.62^a^	3.40^b^
Fe	15.82^b^	20.44^a^	25.49^a^	7.61^c^	27.78^a^	8.82^c^

*The results are shown as the mean of three repetitions (n = 3). OWP, soil sample taken within a radius of 0.5 m; OW, soil sample taken at a distance of 3 m from the oil well. Treatment means separated by different letters are significantly differ (Tukey’s mean separation test, *P* < 0.05)*.

### Functional Diversity of Soils Assessed by CLPP

**FIGURE 2:**
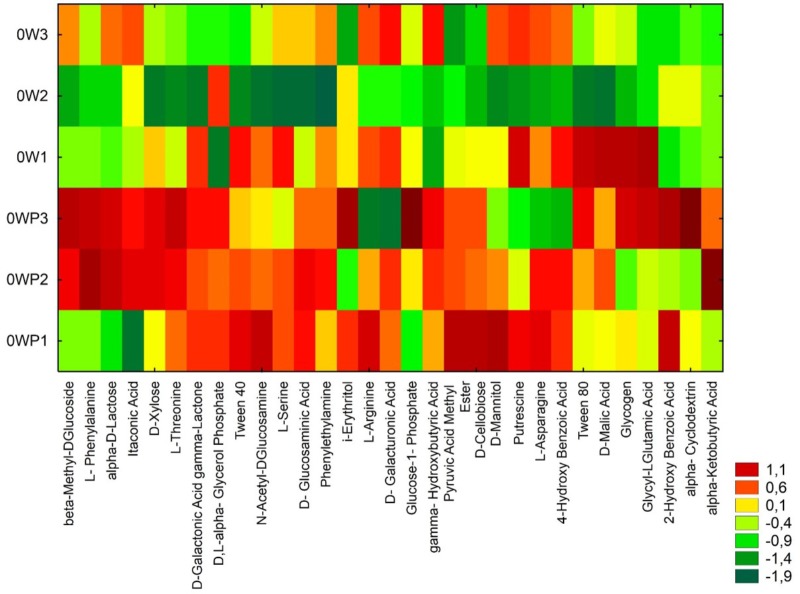
Heatmaps for the carbon utilization patterns of the substrates on the Biolog EcoPlates. Data from soil derived samples incubated for 120 h. OWP, soil sample taken within a radius of 0.5 m; OW, soil sample taken at a distance of 3 m from the oil well.

The soils samples collected directly from oil wells were characterized by higher values on CLPP (**Figure 2**). Heatmaps for the carbon utilization patterns of the substrates located on the Biolog EcoPlates, incubated for 120 h, showed significant differences between soil samples. The highest activity in carbon utilization patterns were observed in soils taken 3 m from the oil wells: OW1, OW2, and OW3 (**Figure 2**). This result might indicate that during the long-term contamination the autochthonic microorganisms adapted to live in this environment and were able to use PAHs as their only source of carbon and energy. Notably, the highest diversities based on Shannon–Wiener indexes were obtained in soils taken directly from oil wells: OWP1 (*H*′ = 3.342), OWP2 (*H*′ = 3.228), OWP3 (*H*′ = 3.282; **Table 4**). A higher richness value (*R* = 30) and average well-color development (AWCD = 1.553) were observed in soil OWP3. In addition, the lowest value of substrate evenness (*E* = 0.965) and Simpson index of diversity (*D* = 0.960) were found in this same soil sample (OWP3).

**Table 4:** Biodiversity indexes.

Sample ID	Shannon (*H*′) _Biolog_	Simpson (*D*) _Biolog_	Richness (*R*) _Biolog_	Evenness (*E*) _Biolog_	AWCD_590_Biolog_
OW1	3.201^b^ ± 0.015	0.983^b^ ± 0.03	27.0^b^ ± 0.10	0.971^b^ ± 0.05	1.125^a^ ± 0.10
OW2	3.046^b^ ± 0.029	0.982^b^ ± 0.02	23.67^c^ ± 0.57	0.963^b^ ± 0.01	0.927^b^ ± 0.02
OW3	3.248^b^ ± 0.044	0.989^b^ ± 0.01	27.66^b^ ± 0.56	0.978^b^ ± 0.06	1.033^a^ ± 0.04
OWP1	3.342^b^ ± 0.013	0.976^b^ ± 0.01	23.66^c^ ± 1.15	0.993^b^ ± 0.01	1.354^a^ ± 0.05
OWP2	3.228^b^ ± 0.015	0.979^b^ ± 0.01	28.33^b^ ± 0.57	0.965^b^ ± 0.02	1.428^a^ ± 0.06
OWP3	3.282^b^ ± 0.016	0.960^b^ ± 0.01	30.00^b^ ± 0.01	0.965^b^ ± 0.05	1.553^a^ ± 0.03

*Changes of functional diversities of bacterial communities in soils long-term contaminated with crude oil, as evaluated by the Shannon–Wiener general diversity index (H′); Simpson index of diversity (D); substrate richness (R); substrate evenness (E) and average well-color development (AWCD_*590*_). Data obtained from the Biolog EcoPlates incubated for 48 h. OWP, soil sample taken within a radius of 0.5 m; OW, soil sample taken at a distance of 3 m from the oil well. Treatment means separated by different letters are significantly differ (Tukey’s mean separation test, *P* < 0.05)*.

**FIGURE 3:**
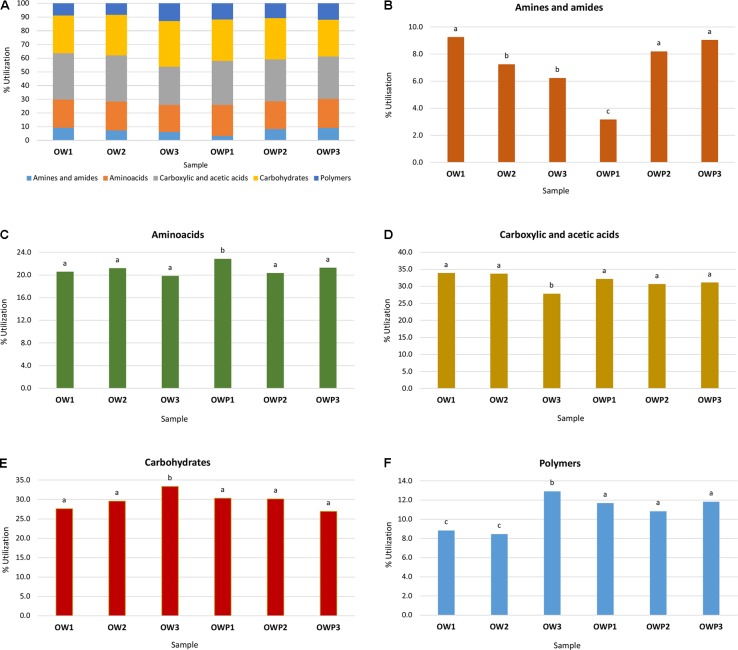
Effect of petroleum impact on microbial community catabolic diversity as evaluated by substrate utilization on Biolog EcoPlates incubated for 120 h. Vertical bars represent the standard error of the mean. Treatment means separated by different letters are significantly different (Tukey’s mean separation test, *P* < 0.05). **(A)** Percent of total carbon source utilization in soil; **(B)** ΣOD_590_ for amines and amides; **(C)** ΣOD_590_ for amino acids; **(D)** ΣOD_590_ for carboxylic and acetic acids; **(E)** ΣOD_590_ for carbohydrates; **(F)** ΣOD_590_ for polymers. OWP, soil sample taken within a radius of 0.5 m; OW, soil sample taken at a distance of 3 m from the oil well.

**FIGURE 4:**
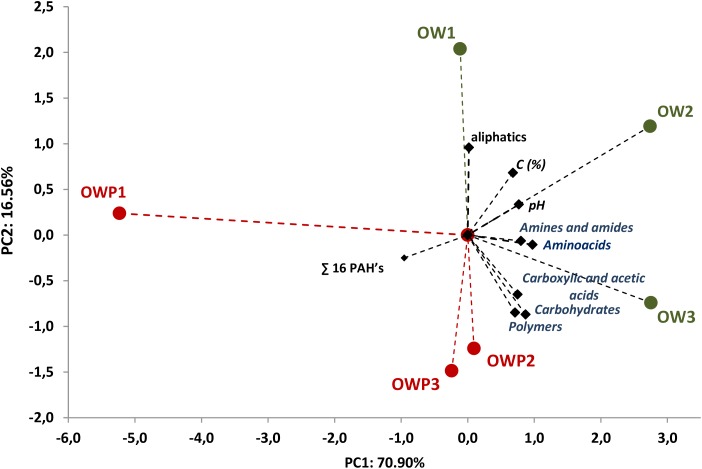
Principal component analysis of PAH content and biodiversity indexes of Biolog EcoPlates data. Data from soil derived samples incubated for 120 h. OWP, soil sample taken within a radius of 0.5 m; OW, soil sample taken at a distance of 3 m from the oil well.

All five main groups of carbon source (carbohydrates, polymers, carboxylic and acetic acids, amino acids, amines and amides) were efficiently used by microorganisms. Amino acids, carbohydrates, carboxylic and acetic acids were groups of compounds which were used by microorganisms much better than the other two groups, polymers and amines (**Figure 3**). Selected indicators of soil microbial diversity (PAH content and Biolog indexes) explained 87.46% of biological variability in soils. Biodiversity indicators obtained from Biolog EcoPlates were strongly correlated with C_org_, pH, Σ16 PAHs and aliphatics (**Figure 4**). This might prove that these hydrocarbons were being degraded by bacteria in the soil of this environment. Based on PCA, 2 different groups of soils were obtained: group I (OW1, OW2, and OW3), and group II (OWP1, OWP2, and OWP3; **Figure 4**). These results confirmed the strong functional bacterial diversity depending on where the sample was collected. Positive correlations were shown between the first and second components of PCA (PCA1 = 70.90%, PCA2 = 16.56%, respectively) and carbon source in the 120 h Biolog EcoPlate incubated soils (**Table 5**).

**Table 5:** Correlation of carbon source with the first (PC1) and second (PC2) components in soils contaminated with crude oil.

Carbon source	Carbon source group	PC1 (70.90%)	PC2 (16.56%)
Pyruvic acid methyl ester	Carbohydrates	-0.96*	-0.09
Tween 40	Polymers	-0.78*	0.51
Tween 80	Polymers	-0.76*	0.33
α- Cyclodextrin	Polymers	-0.68*	-0.52
Glycogen	Polymers	-0.65*	-0.49
D-Cellobiose	Carbohydrates	-0.27	0.34
α-D-Lactose	Carbohydrates	-0.13	-0.70*
β-Methyl-D-glucoside	Carbohydrates	-0.72*	-0.35
D-xylose	Carbohydrates	-0.96*	-0.05
*i*-erythritol	Carbohydrates	-0.07	-0.49
D-mannitol	Carbohydrates	-0.18	0.66*
*N*-acetyl-D-glucosamine	Carbohydrates	0.36	-0.46
D- glucosaminic acid	Carboxylic and acetic acids	-0.54	0.60*
Glucose-1- phosphate	Carbohydrates	-0.42	0.77*
D,L-α- glycerol phosphate	Carbohydrates	0.08	0.63*
D-galactonic acid γ-lactone	Carboxylic and acetic acids	-0.29	0.26
D- galacturonic acid	Carboxylic and acetic acids	-0.17	-0.86*
2-hydroxy benzoic acid	Carboxylic and acetic acids	-0.72*	-0.54
4-hydroxy benzoic acid	Carboxylic and acetic acids	-0.94*	0.15
γ- hydroxybutyric acid	Carboxylic and acetic acids	-0.35	-0.32
Itaconic acid	Carboxylic and acetic acids	-0.32	0.59
α-ketobutyric acid	Carboxylic and acetic acids	-0.81*	-0.43
D-malic acid	Carboxylic and acetic acids	-0.77*	0.45
L-arginine	Aminoacids	-0.81*	0.18
L-asparagine	Aminoacids	-0.05	-0.52
L- phenylalanine	Aminoacids	-0.31	-0.41
L-serine	Aminoacids	-0.88*	-0.06
L-threonine	Aminoacids	-0.42	-0.70*
Glycyl-L-glutamic acid	Aminoacids	-0.77*	0.16
Phenylethylamine	Amines and amides	-0.91*	0.00
Putrescine	Amines and amides	-0.93*	0.03

*Values marked with (^∗^) are statistically significant (P < 0.05), n = 3.*

### Bacterial Genetic Diversity of Soils

There were differences between the metabolic profiles (CLPPs) of the analyzed soils in microbial communities demonstrated by Biolog EcoPlates and the metagenomics approach based on the V3–V4 16S rRNA gene region using NSG. Significant differences in bacterial structure were found between soils taken at different distances from the oil wells. Significant differences were found by PERMANOVA at the family level (*P* = 0.029, *F* = 3.201), genus level (*P* = 0.027, *F* = 2.922, and species level (*P* = 0.034, *F* = 2.563).

**FIGURE 5:**
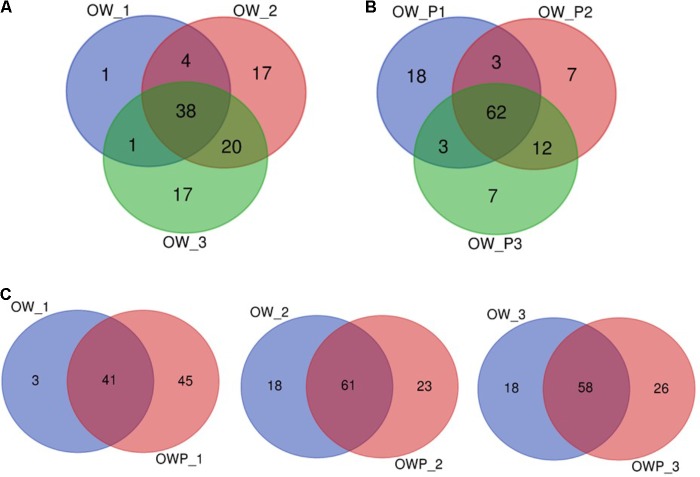
Venn diagram of overlapping bacterial communities from the three oil wells. **(A)** Venn diagram of overlapping bacterial communities from samples OW1, OW2, OW3; **(B)** Venn diagram of overlapping bacterial communities from samples OWP1, OWP2, OWP3; **(C)** Venn diagram of overlapping bacterial communities from samples: OW1 *via* OWP1; OW2 *via* OWP2; OW3 *via* OWP3. OWP, soil sample taken within a radius of 0.5 m; OW, soil sample taken at a distance of 3 m from the oil well.

The Venn diagram for the bacterial communities of the three oil wells based on data from NGS is presented on **Figure 5**. In the case of soils collected directly from oil wells (OWP1, OWP2, and OWP3), 62% of the bacterial microbiome were found to be in common. By contrast, soils collected from a distance of 3 m (OW1, OW2, and OW3) had only 38% in common. This might be explained by the development of completely different groups of bacteria in contaminated soils. Analyzing the distance between soil samples, the overlapping bacteria for oil well no 1 (OW1) was 41%, for OW2 61%, and for oil well no 3 (OW3) 58% (**Figure 5**).

**FIGURE 6:**
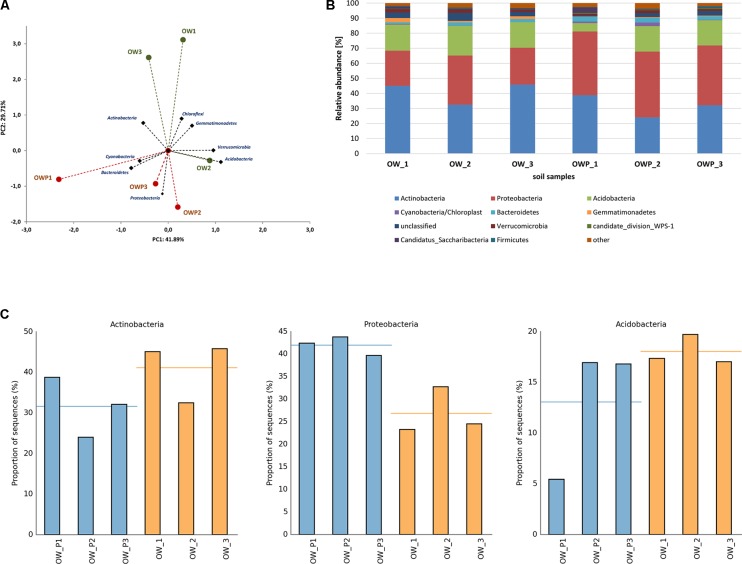
Relative abundance of dominant phyla of bacteria in the different soils (percentage of sequences) based on Next Generation Sequencing. The classifications with less than 1% abundance are gathered into the category “other”. **(A)** Principal component analysis; **(B)** Relative abundance of dominant phyla of bacteria; **(C)** Relative abundance of *Actinobacteria, Proteobacteria*, and *Acidobacteria*. OWP, soil sample taken within a radius of 0.5 m; OW, soil sample taken at a distance of 3 m from the oil well.

A strong correlation between the phyla in different soil samples was observed (**Figure 6A**). These analyses explained 71.6% of the biodiversity in the soils. Based on bacterial phyla as a component of PCA 3 different groups of soils were obtained: group I (OW1 and OW3), group II (OW2) and group III (OWP1, OWP2, and OWP3). Sequences from the V3–V4 16S rRNA gene region, assigned to the reference database, are presented in **Figure 6B**. All the main phyla of bacteria were observed: *Acidobacteria, Proteobacteria, Actinobacteria, Nitrospirae, Bacteroidetes, Chloroflexi, Gemmatimonadetes, Cyanobacteria, Firmicutes, Planctomycetes*, and *Verrucomicrobia* (**Figure 6**). *Actinobacteria* was a dominant phylum in soil taken 3 m from oil well OW1 (45.82%). *Acidobacteria* was the dominant phylum in soils at the same distance from OW2 (19.71%). *Proteobacteria* was the dominant phylum in soil taken directly from oil wells (OWP2, 43.80%; OWP1, 42.42%; OWP3, 39.77%; **Figure 6C**).

**FIGURE 7:**
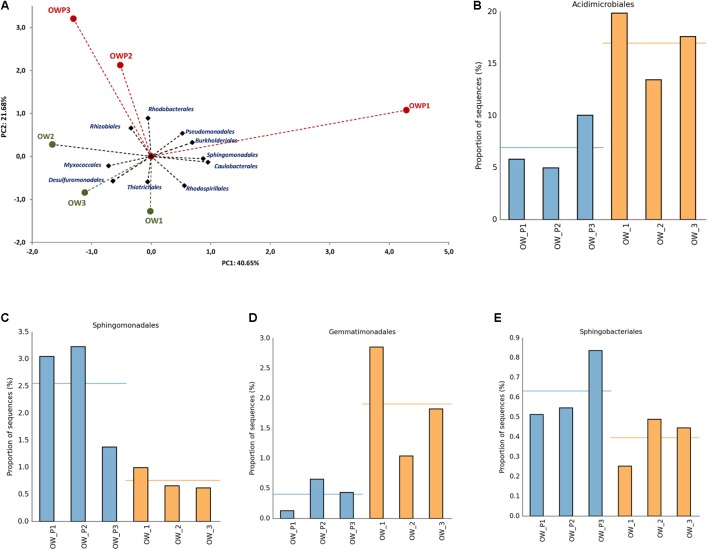
Relative abundance of dominant orders of bacteria in the different soils (percentage of sequences) based on Next Generation Sequencing. The classifications with less than 1% abundance are gathered into the category “other”. **(A)** Principal component analysis; **(B)** Relative abundance of *Acidimicrobiales*; **(C)** Relative abundance of *Sphingomonadales*; **(D)** Relative abundance of *Gemmatimonadales*; **(E)** Relative abundance of *Sphingobacteriales*. OWP, soil sample taken within a radius of 0.5 m; OW, soil sample taken at a distance of 3 m from the oil well.

Principal component analysis using the relative abundance of bacterial orders as a component also showed a strong correlation between different soil samples (**Figure 7A**). This analysis explained 62.33% of biological variability in soils. Based on bacterial orders as a component of PCA, 2 different groups of soils were identified: group I (OW1, OW2, and OW3), and group II (OWP1, OWP2, and OWP3). This analysis confirmed that at the order level there were strong differences in the composition of bacteria in contaminated soil depending on their collection site. *Acidimicrobiales* was a dominant order in soils taken 3 m from the oil wells (OW1, 19.86%; OW3, 17.62%; and OW2, 13.45%). In addition, the second main order was *Actinomycetales* (OW3, 19.35%; OW1, 16.34%; OW2, 13.72%; and OWP, 28.27%; OWP2, 28.27%; OWP3, 10.45%; **Figures 7B–E**).

**FIGURE 8:**
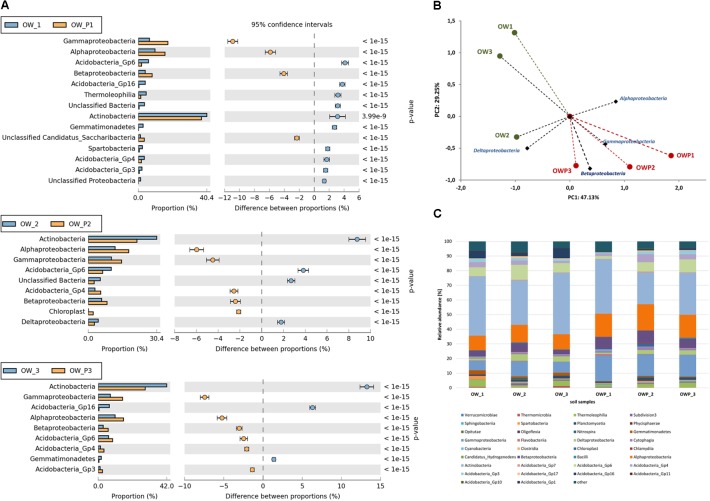
Relative abundance of dominant classes of bacteria in the different soils (percentage of sequences) based on Next Generation Sequencing. The classifications with less than 1% abundance are gathered into the category “other”. **(A)** Relative abundance of dominant classes of bacteria in the different soils (OW1 *via* OWP1; OW2 *via* OWP2; OW3 *via* OWP3); **(B)** Principal component analysis; **(C)** Relative abundance of dominant classes of bacteria. OWP, soil sample taken within a radius of 0.5 m; OW, soil sample taken at a distance of 3 m from the oil well.

Relative abundance of dominant classes of bacteria in the different soils (expressed as percentage of sequences) based on NGS are presented in **Figure 8**. Among the dominating classes found were: *Actinobacteria, Acidobacteria, Solibacteres, Chloracidobacteria, Acidimicrobiia, Thermoleophilia, Cytophagia, Bacilli, Nitrospira, Planctomycetia, Alphaproteobacteria, Betaproteobacteria, Deltaproteobacteria, Gammaproteobacteria* and *Spartobacteria* (**Figure 8A**). PCA showed strong correlations between the classes in different soil samples (**Figure 8B**). This analysis explained 76.38% of the biological variability in soils. *Alphaproteobacteria, Betaproteobacteria, Gammaproteobacteria* were strongly correlated with biological activity in soils taken directly from all oil wells (OWP1, OWP2, and OWP3). *Deltaproteobacteria* were dominated in soil sample OW2. The highest content observed in all soil samples was *Actinobacteria* (OW1, 40,36%; OW2, 30,35%; OW3, 41,97%; OWP1, 37,23%; OWP2, 21,57%; OWP, 28,71%; **Figure 8C**). The second main class was *Alphaproteobacteria* (OW1, 9.86%; OW2, 11.90%; OW3, 10.30%; OWP1, 15.73%; OWP2, 17.88%; OWP, 15.52%). The other classes identified in soils contaminated with petroleum were: *Acidobacteria-5, Flavobacteriia, Saprospirae, Anaerolineae, Chloroflexi, Thermomicrobia, Clostridia, Gemmatimonadetes, Phycisphaerae*, and *Pedosphaerae* (**Figure 8**).

**FIGURE 9:**
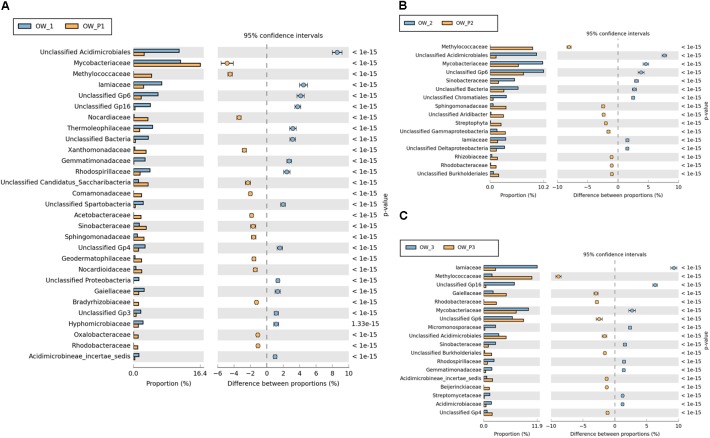
Relative abundance of dominant families of bacteria in the different soils (percentage of sequences) based on Next Generation Sequencing. The classifications with less than 1% abundance are gathered into the category “other”. **(A)** Relative abundance of dominant families of bacteria in samples: OW1 *via* OWP1; **(B)** Relative abundance of dominant families of bacteria in samples: OW2 *via* OWP2; **(C)** Relative abundance of dominant families of bacteria in samples: OW3 *via* OWP3. OWP, soil sample taken within a radius of 0.5 m; OW, soil sample taken at a distance of 3 m from the oil well.

Significant differences in the main family of *Proteobacteria* were observed in contaminated soils depending on where they were collected (**Figure 9**). Some family members belonging to *Alphaproteobacteria* were dominant in soils taken directly from oil wells: *Mycobacteriacea*, *Methylococcaceae*, *Bradyrhizobiaceae, Rhizobiaceae, Rhodobacteraceae, Acetobacteraceae, Hyphomicrobiaceae*, and *Sphingomonadaceae*. The *Streptophyta* and the *Gp6* family were also dominant at this location (**Figure 9**); however, other strains were abundant in soils taken 3 m from the oil wells.

**FIGURE 10:**
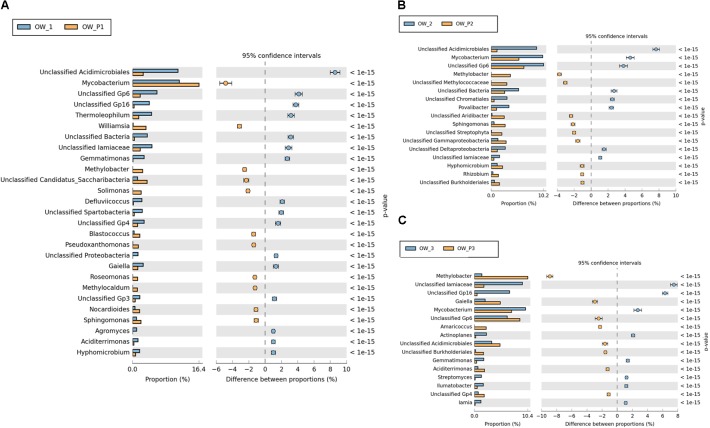
Relative abundance of dominant genera of bacteria in the different soils (percentage of sequences) based on Next Generation Sequencing. The classifications with less than 1% abundance are gathered into the category “other”. **(A)** Relative abundance of dominant genera of bacteria in samples: OW1 *via* OWP1; **(B)** Relative abundance of dominant genera of bacteria in samples: OW2 *via* OWP2; **(C)** Relative abundance of dominant genera of bacteria in samples: OW3 *via* OWP3. OWP, soil sample taken within a radius of 0.5 m; OW, soil sample taken at a distance of 3 m from the oil well.

Valuable information could also be obtained from analysis of bacterial structure at the genus level. However, among the analyzed sequences there are many that were unidentified. There were statistically significant differences in the bacterial structure on the genus level between soils collected directly from the oil well and those collected at a distance of 3 m (**Figure 10**). On the other hand, the analysis of bacterial composition in soil contaminated with crude oil at the species level did not produce good results. Further studies at this level will need to be carried out by sequencing longer DNA fragments. Even so, in the case of species-level analysis, most of the sequenced species are non-cultivated bacteria that have not been classified (**Figure 10**).

## Discussion

Polycyclic aromatic hydrocarbons and other petroleum derivatives have a high potential to accumulate in the soil environment, where they can interfere with the soil’s microbiome (Gałązka and Grządziel, 2016). Bacteria that biological degrade petroleum derivatives often show synergistic effects and are one of the most effective and secure ways of removing hydrocarbons and PAHs from the environment, though the process is lengthy and multistage (Semple et al., 2006). The knowledge of the nature and diversity of bacterial communities in oil fields is still scarce, and their metabolic activities *in situ* largely unknown. There is a great need for research directly at sites of contamination in order to be able to identify and characterize bacteria, both genetically and functionally. Clearly, such studies can benefit from using a combination of molecular-based techniques and functional analysis (Li et al., 2008).

Petroleum compounds are mainly composed of carbon and hydrogen and only a small number of them contain nitrogen (Vazquez et al., 2013; Silva et al., 2015). This causes the C:N ratio to increase significantly in contaminated soil (Sutton et al., 2013). Determining the synergistic interactions, and taking into account the optimum plant-microsymbiont systems, could be the beginning of work on the development of effective and ecologically sound cometabolic systems for treating soils contaminated with petroleum derivatives and trace elements (Nwaichi et al., 2015). de Oliveira et al. (2008) showed statistically differences in the dominant bacterial taxones, in oil samples originating from three reservoirs presenting with distinct levels of biodegradation. The dominant bacterial species were *Rhodococcus* sp., *Acidithiobacillus ferrooxidans*, *Alicyclobacillus acidoterrestris, Bacillus* spp., and *Streptomyces* sp.; as well as finding *Halanaerobium* sp. and *Pseudomonas* sp.

Our studies clearly proved that long-term contamination of soil also induces changes in the bacterial community structure and their metabolic activity, with the emergence of different groups of bacteria. The *Alphaproteobacteria*, *Betaproteobacteria*, *Gammaproteobacteria* were strongly correlated with biological activity in soils taken directly from oil wells (OWP1, OWP2, and OWP3). The *Deltaproteobacteria* was dominated in soil OW2. Some specific families of *Alphaproteobacteria* were dominant in soil taken directly from oil wells: *Bradyrhizobiaceae, Rhizobiaceae, Rhodobacteraceae, Acetobacteraceae, Hyphomicrobiaceae*, and *Sphingomonadaceae*. By contrast other families were found in soil taken 3 m from oil wells. The highest biodiversities based on Shannon–Weaver indexes were obtained in soils taken directly from oil wells.

Bacteria that use PAHs as the only source of carbon include: *Pseudomonas* sp., *Aeromonas* sp., *Bacillus* sp., *Acinetobacter* sp., *Alcaligenes* sp., *Arthrobacter* sp., *Micrococcus* sp., *Mycobacterium* sp., *Sphingomonas* sp., *Rhodococcus* sp., *Flavobacterium* sp., and *Vibrio* sp. (Zhong et al., 2011). In addition, many genera of *Actinobacteria* take part in hydrocarbon degradation, including: *Acinomyces* sp., *Nocardia* sp., *Streptomyces* sp. (Naether et al., 2012). Members from *Bacillus, Rhizobium, Rhodococcus* have also been associated with petroleum and hydrocarbon degradation (Doong and Lei, 2003; Zhong et al., 2011; Sutton et al., 2013). In our study this group was also ostensibly represented. In the study of Yergeau et al. (2012) the dominant phylum of bacteria obtained from soil collected from oil sands mining was *Proteobacteria*, with *Alphaproteobacteria* and *Betaproteobacteria* being the most dominant classes. In addition, *Bacteroidetes, Firmicutes*, and *Chloroflexi* were highly represented. Some long-term oil contaminated samples had relatively more *Betaproteobacteria*, *Epsilonproteobacteria*, and *Deltaproteobacteria* and relatively less of all other groups. This relative increase in *Betaproteobacteria* and *Deltaproteobacteria* was primarily due to 3 taxa, *Rhodoferax*, *Thiobacillus*, and *Smithella*, which also dominated in the control as well. By contrast, sequencing and phylogenetic analyses of Dellagnezze et al. (2016) revealed a bacterial community mostly represented by members of the genera *Petrotoga*, *Bacillus*, *Pseudomonas* and *Rahnella*. The most abundant genera were: *Pseudomonas* (15.2%), *Paenibacillus* (10.8%), *Kocria* (8.7%), *Achromobacter* (6.5%), *Leuconostoc* (6.5%), *Thermicanus* (6.5%), *Petrotoga* (4.4%), *Acinetobacter* (2.2%), and *Bacillus* (2.2%).

Model microorganisms used in the bioremediation process include *Mycobacterium* sp. of genus *Mycobacterium* and *Pseudomonas*. Bacteria of the genus *Mycobacterium* strain PYR-1 decompose all 3-, 4-, and 5-ring PAHs, except for chrysene (Child et al., 2007). In soils contaminated with anthracene, phenanthrene, and pyrene, the microorganisms degrading PAHs are mostly of the genus *Pseudomonas* (Doong and Lei, 2003), including *P. fluorescens*, *P. putida*, and *P. paucimobilis.* Also among these is *P. stutzeri*, which binds free nitrogen in the presence of various substrates which are its source of carbon and energy.

The isolation and characterization of new species and strains capable of using PAHs as the sole carbon and energy sources, has focused on their isolation from contaminated soils, and has included: *Mycobacterium pallens* sp. nov., *M. crocinum* sp. nov., *M. rutilum* sp. nov., *M. rufum* sp. nov., and *M. aromaticivorans* sp. nov. These strains were described as new species of bacteria (nov.) capable of degrading PAHs (Zhong et al., 2011). In recent times, electron acceptors other than oxygen have been introduced into the environment, stimulating anaerobic processes of decomposition of organic pollutants. Under these conditions, microorganisms can digest most aliphatic and aromatic hydrocarbons. They are completely or partially degraded by bacteria that are: denitrifying, sulfate-reducing, iron or molybdenum nitrogen-reducing, or methanogenic (Chen et al., 2005). Bacteria from genera such as *Azoarcus* (*A. toluvorans, A. toluclasticus, A. evansii*) *Paracoccus, Ochrobactrum, Thaurea, Burkholderia kururiensis, Bradyrhizobium*, and *Mezorhizobium* can decompose various benzene compounds during denitrification. The processes of biodegradation and detoxification of phenols and *trans*-dihydrodiols can occur simultaneously with the transformation of sulfates, glucuronic acid, glucose or xylose. Some strains of the genus *Pseudomonas* (strain T and K172) can oxidize toluene and m-xylene under anaerobic conditions.

In our study among the dominating classes found were: *Acidobacteria, Solibacteres, Chloracidobacteria, Acidimicrobiia, Actinobacteria, Thermoleophilia, Cytophagia, Bacilli, Nitrospira, Planctomycetia, Alphaproteobacteria, Betaproteobacteria, Deltaproteobacteria, Gammaproteobacteria*, and *Spartobacteria*. The presence of these bacteria in the contaminated soil depended significantly on both the contamination site and the petroleum derivatives found in the soils. These bacteria were very sensitive indicators of soil quality in contaminated soils.

The bacterial composition in the soil collected from the oldest oil wells in Węglówka was not typical for soils contaminated with petroleum. Soil structure is the main determinant of genus composition; however, for this study all the soils belonged to one type. Long-term contamination has resulted in adaptation to the conditions of contamination by select groups and types of bacteria. Notably, the presence of such species as *Rhizobium leguminosarum* and *Azospirillum brasilense* confirmed the high bioremediation activity of these soils. *Azospirillum* spp. has been shown to be involved in bioremediation of soils artificially contaminated with PAHs (Gałązka et al., 2012; Gałązka and Gałązka, 2015).

Selection of bacteria capable of bioremediating PAHs could be extremely useful in determining the role of specific microorganisms in soil. It is essential to understand the functional and structural diversity of bacterial at a contaminated site. Only with this knowledge can one contribute to the proper management and cleaning of such areas.

## Conclusion

(1) The results of our study indicated significant differences in both genetic and catabolic bacterial diversity in soils long-term contaminated with petroleum. The soil samples collected directly from oil well were characterized by higher biodiversity. These soils were also characterized by a different structural diversity and some different groups of bacteria compared to the soils taken at a distance of 3 m from the oil wells.
(2) The use of two research methods (the bacterial NGS and CLPP techniques) contributed to a better understanding of the functional and structural diversity of bacteria in contaminated soils.
(3) The content of different trace elements and PAHs in the soil samples contributed to the differentiation of the bacterial composition in the soils. Individual trace elements could be involved in the activation of several enzymes which could aid cometabolic degradation of PAHs. This might indicate that during long-term contamination autochthonic microorganisms have adapted to live in this environment and were able to use PAHs as their only source of carbon and energy.
(4) Determining the bacterial structure and function in contaminated soil is the basis for further studies to identify active bacteria strains in bioremediation. Future studies are needed to select bacterial strains, particularly active in PAH degradation, which could be used in active management of bioremediation processes.
(5) Some family members belonging to *Alphaproteobacteria* were dominant in soils taken directly from oil wells: *Myco bacteriacea, Methylococcaceae, Bradyrhizobiaceae, Rhizo biaceae, Rhodobacteraceae, Acetobacteraceae*, *Hypho- *microbiaceae*, and *Sphingomonadaceae.** This family of bacteria they can be a good indicator of soils petroleum contaminated.

## Author Contributions

AG conceived and designed the experiments. JG, RG, AU-J, JS, and BS participated in writing, statistical, graphical elaboration, revising, and final approval of the manuscript. All the authors accept accountability for all aspects of the work.

## Conflict of Interest Statement

The authors declare that the research was conducted in the absence of any commercial or financial relationships that could be construed as a potential conflict of interest.
